# Balanced Functional Module Detection in genomic data

**DOI:** 10.1093/bioadv/vbab018

**Published:** 2021-09-16

**Authors:** David Tritchler, Lorin M Towle-Miller, Jeffrey C Miecznikowski

**Affiliations:** Department of Biostatistics, University at Buffalo, Buffalo, NY 14260, USA; Biostatistics Division, University of Toronto, Toronto, ON M5S 1A1, Canada; Department of Biostatistics, University at Buffalo, Buffalo, NY 14260, USA; Department of Biostatistics, University at Buffalo, Buffalo, NY 14260, USA

## Abstract

**Motivation:**

High-dimensional genomic data can be analyzed to understand the effects of variables on a target variable such as a clinical outcome. For understanding the underlying biological mechanism affecting the target, it is important to discover the complete set of relevant variables. Thus variable selection is a primary goal, which differs from a prediction criterion. Of special interest are functional modules, cooperating sets of variables affecting the target which can be characterized by a graph. In applications such as social networks, the concept of balance in undirected signed graphs characterizes the consistency of associations within the network. This property requires that the module variables have a joint effect on the target outcome with no internal conflict, an efficiency that may be applied to biological networks.

**Results:**

In this paper, we model genomic variables in signed undirected graphs for applications where the set of predictor variables influences an outcome. Consequences of the balance property are exploited to implement a new module discovery algorithm, balanced Functional Module Detection (bFMD), which selects a subset of variables from high-dimensional data that compose a balanced functional module. Our bFMD algorithm performed favorably in simulations as compared to other module detection methods. Additionally, bFMD detected interpretable results in an application using RNA-seq data obtained from subjects with Uterine Corpus Endometrial Carcinoma using the percentage of tumor invasion as the outcome of interest. The variables selected by bFMD have improved interpretability due to the logical consistency afforded by the balance property.

**Supplementary information:**

Supplementary data are available at *Bioinformatics Advances* online.

## 1 Introduction

Clinical outcomes of interest, such as risk factors, disease diagnosis or treatment success may be better understood by identifying associated genes within high-dimensional genomic datasets. Additionally, these associated genes should be associated with one another, such that they may form a ‘module’ or ‘pathway’ that coordinate together to ultimately control the targeted outcome of interest. We denote these outcome-associated sets of variables as ‘functional modules’. Many variable selection methods currently exist to identify functional modules, but these existing functional module detection methods fail to consider the concept of ‘balance’ frequently used in other applications such as social networks. Balance assures no contradictions exist between variables within the functional module. For example, suppose the expression of a certain module gene promotes an increase in the outcome variable while another gene in the same module suppresses it; balance says that the two genes must be negatively correlated, otherwise they would conflict with respect to their effect on the outcome.

Since observed correlations in a balanced signed graph can be composed to reflect all path signs in the underlying graph, we can use the observed correlations to determine if a subset of the high-dimensional set of variables satisfies the balance property. This leads to our new module discovery algorithm, balanced Functional Module Detection (bFMD). The bFMD method detects which variables are contained within the module; after they are selected, various existing analytical methods may be used on the detected module variables for obtaining the graphical structure. For example, modeling predictors as an undirected graph whose nodes influence the target is related to traceable regressions (Wermuth, 2012). Traceable regression analyzes ordered causal blocks of variables and identifies direct and indirect effects among the variables. In our simple scenario, there are two blocks, the block consisting of only the target and a block of potential predictors. The traceable regression analysis of our semidirected graph would be partitioned into an undirected graphical model analysis (e.g. Edwards, 2000; Edwards *et al.*, 2010) of the predictors and individual regressions of the target on each predictor (Wermuth and Cox, 2013). Dimension reduction prior to a fully detailed traceable regression analysis is desirable (Bourgon *et al.*, 2010; Towle-Miller *et al.*, 2020), and the set of variables selected should make causal sense. Thus, initial analysis by bFMD can expand the application of existing methods to high-dimensional data and yield more interpretable models.

We emphasize that the goal of our method is variable selection, not prediction. The outcome variable is modeled to constrain the variable selection for the logical consistency of signs. Optimizing prediction and optimizing variable selection are not the same thing. For selection and scientific understanding, we want all the relevant variables, while prediction can stress parsimony. While a good prediction equation will favor functional variables, it does not require that all network variables are included to provide the basis for a biological explanation. A good example is the lasso (Tibshirani, 1996) which eliminates correlated variables, which might cloud understanding the biological mechanism. The object of our analysis to provide the scientist with a parcel of variables that allow a complete picture of the underlying biology, a set with no obvious self-contradictions in causing the response.

Our development starts with a weighted signed graph model for predictor variables where the edge weights can be positive or negative to describe direct or inverse covariation. A univariate outcome variable *Y* and associated directed signed edges from the predictors are added to the graphical model, resulting in a semidirected graph which we refer to as a functional module. In Section 1.1.3, we introduce the concept of balance in an undirected graph as described in Definition 1.1, and Definition 1.2 extends the balance concept to semidirected graphs which are of primary interest. Using the theory of Gaussian graphical models, we relate observed covariances to the underlying functional module and use them to form a matrix where the submatrix corresponding to the functional module must be positive in order to satisfy balance. Finally, we introduce our module discovery algorithm bFMD, a new method for module discovery algorithm-based Perron theory of positive matrices (Meyer, 2000, ch. 8) and using the sparse positive principal component algorithm from Sigg and Buhmann (2008) to identify the balanced module.

### 1.1 Preliminaries

#### 1.1.1 Weighted undirected graphs

Let G=(X,E,w) denotes a weighted, undirected graph with node set $X=\{x_{1},x_{2},\ldots ,x_{p}\}$; edge set *E* and weight function *w*: E→R. Note that *G* is a signed graph since the weights can be either negative or non-negative. To alleviate cumbersome notation, we will interchangeably denote *x_i_* as *i* when considering *x_i_* as a graph node. We further denote the weight of an *i—j* edge as *w_ij_*.

We assume that a path γ:i→j of length *m* between nodes *i and j* creates an ordered sequence of non-repeated adjacent indices of the form $\gamma =((i,k_{1}),(k_{1},k_{2}),\ldots ,(k_{m},j))\in E^{m}$, where *k_l_* corresponds to the intermittent indices between *i and j*. We calculate the path weight as $w^{\gamma }=w_{ik_{1}}w_{k_{1}k_{2}}\cdots w_{k_{m}j}$. Note that the resulting sign of $w^{\gamma }$ will be negative if and only if there is an odd number of negative edges along the path. We additionally define a ‘walk’ as a path with repeated nodes, and a ‘cycle’ as a path where *i *=* j*.

Further note that we may consider the node set *X* as a vector or *x_i_* as a graph node/variable where appropriate when discussing the statistical properties throughout this manuscript.

#### 1.1.2 Functional modules

We informally define a ‘functional module’ to be a set of coordinated variables $X=\{x_{i};i=1,2,\ldots p\}$ that influence an outcome variable *Y*. Each variable in *X* influences *Y* either directly or indirectly by operating through other variables in the set *X*. Such modules that help describe the response *Y* can be profitably described by an underlying weighted undirected graph G=(X,E,w) under special properties.

*G and Y* create a semidirected graph G∪Y where each node in *G* is a potential predictor of *Y*. The directed portion of G∪Y corresponds to the edges connecting from *G* to *Y*, and the undirected portion of G∪Y corresponds to the edges that connect variables within *X* but not *Y*. We represent a semidirected path α:i→Y in G∪Y as two segments: an undirected path γ:i−j from *i* ∈G terminating in some *j* ∈G followed by a directed edge from *j* to *Y*. We define the weight of the path *α* from *i* to *Y* along *γ* to be $w^{\gamma \cup Y}=w^{\gamma }\cdot \beta_{Y|j.X/i,j}$, where $w^{\gamma }$ represents the path weight from *i* to *j* in *G* and $\beta_{Y|j.X/i,j}$ represents the partial regression coefficient of *j* in the regression model for response *Y* including all variables in *X except x_i_*. Note that $\mathrm{sign}(\alpha )=\mathrm{sign}(w^{\gamma })\times \mathrm{sign}(\beta_{Y|j.X/i,j})$ since $\beta_{Y|j.X/i,j}$ characterizes the directed edge, and the path weight equals $\beta_{Y|j.X/j}$ when a path consists of only a directed edge from *x_j_* to *Y*.

#### 1.1.3 Balance in functional modules

Building on the definitions described in previous sections, we further require the signed graph *G* to be *balanced* (Harary, 1953). A characterization of balance that best results in desired properties of a functional module is described in Definition 1.1. For example, if *i* is related to *j* by a path of module elements, no other *i—j* paths in the network should exist with opposing signs. This balanced mechanism ensures no conflicts, and it greatly improves the interpretability in biological systems by identifying sets of variables that operate in this cooperative fashion.

Definition 1.1  **Balanced Signed Graph**. *A signed graph G is‘balanced’if and only if for every two nodes of G, all paths joining them have the same sign (**Chartrand*, 1977*, Ch. 8; Harary, 1953).*

To model our functional network, we extend the concept of balance to semidirected graphs by requiring all semidirected paths α:i→Y to have the same sign, which results in a similar interpretation to balance within *G*. More precisely, we require a functional network *G* ∪*Y* to have the properties described in Definition 1.2. In summary, *G* is a *module* or network (by the second condition) with no internal conflict (by the first and fourth conditions), and the module affects *Y* (by the third condition).

Definition 1.2  **Balanced Functional Network (**G∪Y**).** *G* ∪*Y is a‘balanced functional module’when the following are satisfied.*

1. *G is balanced.*
2. *G is connected: at least one path connecting each pair of elements within G exists.*
3. *Every node of G is connected to Y by a semidirected path with at least one directed edge.*
4. *For a given node* i∈G*, all i—Y paths have the same sign.*

In Theorem 1.1, we establish a consequent constraint on triplets of paths in a balanced functional network. Note the Supplementary Appendix contains proofs for all subsequent theorems.

Theorem 1.1 *For balanced functional network* G∪Y*, let* α∪Y*equal a semidirected* i→Y*path, γ equal an i—j path in G, and* τ∪Y*equal a semidirected* j→Y*path. Then* $w^{\alpha \cup Y}\cdot w^{\gamma }\cdot w^{\tau \cup Y}>0$.

### 1.2 Statistical model

We represent *G* statistically as a Gaussian undirected graphical model with a nonsingular concentration matrix $\Theta =\Sigma^{-1}$. The properties of Θ will mimic those from previous studies (Anufriev and Panvhenko, 2015; Sulaimanov and Koeppl, 2016; Wermuth, 1980; Whittaker, 2009). Specifically, we denote the diagonal of Θ as $D=\mathrm{diag}(\Theta_{11},\Theta_{22},\ldots ,\Theta_{pp})$, where $d_{i}=\Theta_{ii}=\mathrm{Var}{\left(x_{i}|X/i\right)}^{-1}$ denotes the reciprocal of the partial variance of *x_i_*. Additionally, letting *I* denote an identity matrix of appropriate size, the adjacency matrix $A=I-D^{-1/2}\Theta D^{-1/2}$ contains partial correlations with zeros on its diagonal, making W=I+A a matrix of partial correlations. Note that the off-diagonal elements of *A* correspond to the edge weights from graph *G*. From this, we see that the partial covariance matrix is $C=D^{-1/2}WD^{-1/2}$, and the matrix of least square partial regression coefficients is $B=CD=D^{-1/2}WD^{1/2}$. The covariance matrix $\Theta^{-1}=\Sigma =(\sigma_{ij})$ relates to the adjacency matrix *A* of the graph *G* through the expression

(1) $$\begin{array}{cc} \Sigma & =D^{-1/2}{\left(I-A\right)}^{-1}D^{-1/2}\\ & =D^{-1/2}{\left(D^{-1/2}\Theta D^{-1/2}\right)}^{-1}D^{-1/2}\\ & =D^{-1/2}(D^{1/2}\Sigma D^{1/2})D^{-1/2}. \end{array}$$

The covariance structure of *G* can be described in terms of a closure matrix $G^{*}$ (Meyer, 2000, ch. 7; Sulaimanov and Koeppl, 2016). Powers of the adjacency matrix sum walk in *G* of a specific length, e.g. $A_{ij}^{k}$ equals the sum of all *i—j* walk weights in *G* of length *k*. Then the Neumann series $\Sigma_{m=1}^{\infty }A^{m}$ quantifies the connectivity of *G* and converges to ${\left(I-A\right)}^{-1}=D^{-1/2}\Sigma D^{-1/2}$ (Sulaimanov and Koeppl, 2016). The off-diagonal elements of Σ are then given by $\Sigma_{ij}=d_{i}^{-1/2}w_{ij}^{*}d_{j}^{-1/2}$, where each $w_{ij}^{*}$ corresponds to a weight in the closure $G^{*}$ of *A*, making Σ_*ij*_ the scaled sum of weights of all *i—j* walks in *G*. When the spectral radius of *A*, denoted σ(A), is less than 1 then the Neumann series converges and $\mathrm{sign}(\Sigma_{ij})=\mathrm{sign}(w_{ij}^{*})$. It may be shown then when all row sums of the absolute values in *A* are less than 1, then σ(A)<1. This is interpreted as moderate conditional dependence among the variables since *A* consists of partial correlations (Anufriev and Panvhenko, 2015).

Balance is defined in terms of paths in the literature, while the preceding development describes Σ using walks. To bridge this disconnect, we require Lemma 1.2.

Lemma 1.2 *For a balanced graph G, the sign of an i—j walk is the sign of every i—j path in G*.

Note that Lemma 1.2 implies that every *i—j* walk contains the same sign because according to Definition 1.1, every *i—j* path must have the same sign to be balanced. An immediate consequence is shown in Theorem 1.3.

Theorem 1.3 *For a balanced graph G and corresponding covariance matrix* $\Sigma =(\sigma_{ij})$*, if we assume moderate conditional dependence then the sign of σ_ij_ is equivalent to the sign of every i—j path in G*.

We extend the above concept to the semidirected case in Theorem 1.4.

Theorem 1.4 *For a balanced functional network* G∪Y*with G satisfying the conditions ofTheorem 1.3*:

1. *Cov*(*i*, *Y*) is a positively weighted sum of all *i—Y* walk weights
2. The sign of *Cov*(*i*, *Y*) equals the sign of every *i—Y* path.
3.
Cov(i,Y)≠0 .

## 2 Methods

### 2.1 Identification of module elements

We use Theorems 1.1, 1.3 and 1.4 to guide the discovery of subsets within *X* which influence *Y* and behave as a balanced graph. To this end, we define the matrix *M* in Theorem 2.1, and balanced functional modules will appear as positive submatrices of this matrix.

Theorem 2.1 *If the graph* Cov(i,Y)≠0G∪Y*within X is a balanced functional module, then*

(2) M=diag(Cov(X,Y))Var(X)diag(Cov(X,Y))

*is a positive matrix (i.e. all elements of M are* > 0).

Note that the (*i*, *j*)th element of *M* equals $\mathrm{Cov}(x_{i},Y)\mathrm{Cov}(i,j)\mathrm{Cov}(x_{j},Y)$, making it large when *i and j* are highly correlated with each other (i.e. in the same module) and with *Y* (i.e. both functional). Also, if *i and j* are uncorrelated, or either is unrelated to the outcome, the corresponding matrix element will be zero. Thus, *M* will highlight sets of interrelated variables and also incorporate a functional constraint. Theorem 2.1 justifies a mechanism for identifying the subset of module elements by analyzing a matrix similar to (2) by using the full data collected. The data collected will contain a high-dimensional set of variables, and the module *M* will be a subset of the larger matrix. If we compute the matrix $M^{*}=\mathrm{diag}(\mathrm{Cov}(X^{*},Y))\Sigma (X^{*})\mathrm{diag}(\mathrm{Cov}(X^{*},Y))$ for the entire set of predictors $X^{*}$, then the balanced functional module *M* will appear as a positive submatrix of $M^{*}$. The nonzero elements correspond to correlated variable pairs that are additionally associated with *Y*. When elements equal zero, they fail to satisfy all three conditions. Since the ordering of the variables is arbitrary, we can write $M^{*}$ as a matrix composed of zeros with a positive upper diagonal block *M* corresponding to the functional module. We then model the observed data matrix as $W=M^{*}+E$, where *E* is a matrix of mean zero errors. In other words, *W* is a noisy, observable version of the unobserved $M^{*}$, and *W* inherits the interpretation given above for *M*. Thus, the identification of a positive submatrix within *W* can approximate the functional module *M*.

To depict this, note that *W* can be written as the Hadamard product

(3) $$E(W)=\mathrm{Var}(X^{*})\cdot \mathrm{Cov}(X^{*},Y)\mathrm{Cov}{\left(X^{*},Y\right)}^{T}),$$

where the operator · denotes element-wise multiplication. The left matrix, $\mathrm{Var}(X^{*})$, contains the information on the modular structure, and the right matrix, $\mathrm{Cov}{\left(X^{*},Y\right)}^{T}$, is positive when the variables corresponding to that element are correlated with each other and with *Y*. Taken from Miecznikowski *et al.* (2016), Figure 1 visually depicts the formation of the Hadamard product (3) for three simulated modules, one of which is functional.

**Fig. 1.**
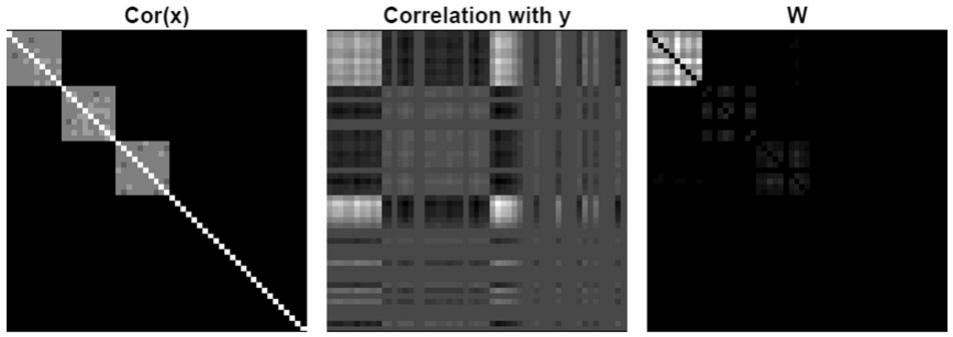
The formation of $W=\mathrm{Cov}(X)\cdot (\mathrm{Cov}(X,Y)\mathrm{Cov}{\left(X,Y\right)}^{T})$. The leftmost matrix is *Cov*(*X*), the middle matrix is $\mathrm{Cov}(X,Y)\mathrm{Cov}{\left(X,Y\right)}^{T}$ and the rightmost matrix is their element-wise product *W*

### 2.2 Balanced Functional Module Detection

Assume a high-dimensional set of variables $x_{i};(i=1,\ldots ,T)$ where *p* variables from this set belong to a functional module *M* and T≫p. Using Theorem 2.1, we first estimate $M^{*}=\mathrm{diag}(\mathrm{Cov}(X^{*},Y))\mathrm{Var}(X^{*})\mathrm{diag}(\mathrm{Cov}(X^{*},Y))$ for the full set of *T* variables. By the Perron Theorem for positive matrices (Meyer, 2000, ch. 8), the submatrix *M* contains the largest absolute eigenvalue λ>0, with corresponding positive eigenvector *v*. Subsequently, $M^{*}$ contains the largest absolute eigenvalue λ>0 with sparse eigenvector (v,0), where 0 denotes a vector of *T—p* zeros.

Our task is to extract a sparse eigenvector from matrix *W*, a noisy version of $M^{*}$, where the eigenvector is composed of either zeros or positive values. Simply put, the goal is to identify variables corresponding to large values within *W*, and these variables corresponding to large values within *W* will be considered part of the module. Variables with large values within *W* will be identified using sparse principal component analysis (sPCA; Sigg and Buhmann, 2008) which returns an eigenvector of size *T* where element *i* within the eigenvector corresponds to variable *i*. Large values within the eigenvector suggest that the variable has larger values within *W*, and sPCA will truncate small values within the eigenvector to zero. The nonzero eigenvector components are used to identify the module *M*. We introduce a novel method that follows naturally from the proceeding theoretical development. Note that an alternative to the more explanatory form (2) is $W=Z^{T}Z$, where $Z=\mathrm{Hdiag}(\mathrm{Cov}(X^{*},Y))$ and *H* is the column-centered version of $X^{*}$. As summarized in Algorithm 1, we calculate the transformed data matrix *Z*, recast the analysis as an sPCA of *Z* using the algorithm from Sigg and Buhmann (2008) and then use the nonzero loadings from the sPCA to select module elements.

The sparse principal component method from Sigg and Buhmann (2008) specifies sparsity by inputting the number of nonzero elements, *k*. We calculate the positive eigenvector for all feasible *k* (e.g. module sizes), and we select the sparsity setting *k* resulting in the optimally balanced solution. Additional details are described in the Supplementary Appendix.

Algorithm 1 Balanced Functional Module Detection

**Require:** $X^{*}$ , *Y* $H=X^{*}-\mathrm{colmeans}(X^{*})⊗1$, where **1** equals a column vector of 1’s

$Z=H\times \mathrm{diag}(\mathrm{Cov}(X^{*},Y))$

$W=Z^{T}Z$

q= eigenvector from sPCA on *W*

Module elements = $\left\{i:q_{i}\neq 0\right\}$

### 2.3 Evaluating balance

We provide a metric for evaluating the balance of a computed module based on an equivalent characterization of balance in an undirected signed graph: *a signed graph G is balanced if its nodes can be partitioned into subsets A and B where all edges within the subsets are positive and all edges connecting the nodes in different sets are negative* (Chartrand, 1977, Ch. 8).

Lemma 2.2 *With balanced A and B as defined above, all paths in the signed graph G connecting nodes in the same set are positive and all paths connecting a node in A to a node in B are negative.*

For balanced semidirected graphs, we characterize correlations in Theorem 2.3.

Theorem 2.3 *For a balanced functional network* G∪Y*, G can be partitioned into two sets of variables A and B such that elements of A are positively correlated, elements of B are positively correlatedand correlations between A and B are negative. All elements of the same set have correlations with Y of the same sign, which is opposite for the two sets.*

Theorem 2.3  implies that for module elements *x_i_ and x_j_*, the sign of $z_{i,j}=\mathrm{Cov}(x_{i},y)\times \mathrm{Cov}(x_{j},y)$ equals the sign of $\mathrm{Cov}(x_{i},x_{j})$. That is, if *x_i_ and x_j_* reside in the same set, their correlation is positive and $\mathrm{sign}(\mathrm{Cov}(x_{i},y))=\mathrm{sign}(\mathrm{Cov}(x_{j},y))$. If *x_i_ and x_j_* reside in different sets, their correlation is negative and $\mathrm{sign}(\mathrm{Cov}(x_{i},y))\neq \mathrm{sign}(\mathrm{Cov}(x_{j},y))$. Thus, we flatten the upper diagonal elements of $Z=(z_{i,j})$ and Σ into corresponding vectors of length n(n−1)/2 and examine the agreement between the signs of the z-vector and the signs of the correlation vector. This can be viewed as the measurement of inter-rater reliability of the two signs. Cohen’s Kappa measures agreement while considering the possibility of chance. A Cohen’s Kappa value less than or equal to 0 corresponds to no agreement/disagreement, and values greater than 0 correspond to agreement with perfect agreement equal to 1. We use Cohen’s Kappa to judge whether the sparse selection has captured agreeable variables according to Theorem 2.3, and hence whether they form a *balanced* functional module.

We note that the theorem shows that in the case of genetic effects, promotors and suppressors are unambiguously classified by the sign of the simple correlation with outcome.

## 3 Results

### 3.1 Simulation

#### 3.1.1 Simulation model

Our method applies to arbitrary module topologies. Our algorithm and its application are independent of the underlying network topology. It is just used to select a causally consistent set of relevant variables. We will simulate its use in the specific case a Single Input Module (SIM), which consists of a set of genes controlled by a single transcription factor (Milo *et al.*, 2002). Experimental evidence shows that SIMs occur frequently (Lee *et al.*, 2002; Milo *et al.*, 2002). For example, consider an SIM represented by the linear model for gene expression

(4) $$x_{i}=\pi_{i}\beta x_{0}+\varepsilon_{i},i=1,\ldots ,t$$

(5) $$x_{0}=\varepsilon_{0},$$

where $\beta >0,\;\pi_{i}\in \{-1,1\}$ and the $\varepsilon_{i},\;i=0,1,\ldots ,t$ are independent errors with mean 0 and variance $\sigma_{\varepsilon }^{2}$. The covariance of all pairs of genes in this system is nonzero. The covariation among the *t* observed module genes is driven by a latent unobserved *hub*, *x*_0_. We model the functional aspect of the pathway by letting the hub *x*_0_ determine an outcome variable *Y* by the regression function

(6) $$Y=\alpha x_{0}+\delta ,$$

where α>0 without loss of generality. Theoretical details on the covariance structure are described in the Supplementary Appendix.

**Fig. 2.**
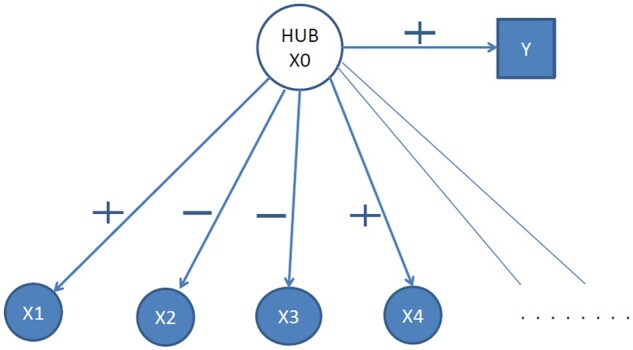
The graph of an SIM. The hub *x*_0_ is an unobserved latent factor that relates to outcome *Y* while $x_{1},x_{2},\ldots ,$ are the observed variables within the functional module. The balance property is demonstrated by tracing paths between nodes

Figure 2 summarizes the components of an SIM. Tracing paths (Wright, 1934) in Figure 2 show that an SIM is a balanced functional module. All predictor nodes positively correlated with *Y* are positively correlated with each other, and similarly for predictor nodes that are negatively correlated with *Y*. The path connecting a pair of nodes is negative if they have differing correlation with *Y*. Consequently, this simple latent variable model commonly used to illustrate biological phenomenon is actually a balanced module.

#### 3.1.2 Simulation settings

We simulated data containing 5 SIM modules with 20 nodes each and a set of 1000 irrelevant nodes, Δ. Only one of the modules is functional as per (6), and the other four are uncorrelated with the outcome variable *Y*. We define signal-to-noise ratio (SNR) to be the ratio of $\alpha \sigma_{\varepsilon }$ to $\sigma_{\delta }$, making SNR a ratio of the square root of the variance component in *Y* attributed to the hub and the variance component in *Y* attributed to the noise. We set model parameters to produce a specified SNR (SNR∈{.3,.5}) and intramodular correlation ($r_{m}\in \{.1,.2,.3,.4,.5,.6,.7\}$), for sample sizes of 100 and 400. We additionally included five independent nodes with SNR equivalent to the hub node, for a total of 1105 nodes. The five independent nodes represent nonmodular influences on the outcome, which realistically may be present in data.

We compare our method (bFMD) to three others. The first (W) was given by Miecznikowski *et al.* (2016) and starts with the matrix *W* defined previously. They set all negative elements of the matrix *W* to zero since the negative elements will correspond to unbalanced node pairs. The transformed matrix will then be matrix of elements corresponding to theoretical zeros and a highly positive block. Since the highly positive block will have a maximal sparse positive eigenvector according to the Perron Theorem, they use it to identify the module variables. The theoretical eigenvector has *p* positive elements and *T—p* zeros, so they partition their approximate eigenvector elements into two clusters by k-means and take the cluster with a higher mean to be the module. This maneuver models sparsity only indirectly so their method does not require specifying a sparsity parameter.

We also contrast our approach with Weighted Correlation Network Analysis (WGCNA), which is a two-step strategy of cluster analysis (modularity) followed by evaluating the identified clusters for the average correlation of the cluster members with the outcome variable (functionality; Langfelder and Horvath, 2008). Like bFMD and W, WGCNA obtains sets of interrelated variables, but in contrast, the functional information captured by the individual gene-outcome associations is not incorporated in the search for clusters at the first step. The basic approach can be implemented in a variety of ways by varying the similarity metric and the clustering method used, and it has been extended and highly developed (Horvath 2011; Zhang and Horvath, 2005) as part of a broad approach to network analysis. To implement it for our simulation study, we expressed similarity as absolute correlation and used partition around medoids (PAM) for the clustering method (Reynolds *et al.*, 2006). The clustering methods were set to obtain six clusters: one for the functional cluster, four for the nonfunctional clusters and an additional cluster to capture other nodes. However, identifying the true number of clusters is difficult in practice. We computed the average significance for predicting *Y* for the genes in each cluster (the module significance), and the functional module was taken to be the most significant cluster.

In related work with a different objective, supervised principal components (SPC; Bair *et al.*, 2006) finds components composed of genes that are predictive of the outcome. In the first step, SPC selects a set genes highly associated with the outcome. Principal components are then computed for just those genes, and the resulting components are used for prediction. The number of selected genes is tuned using cross-validation to minimize the estimated out-of-sample prediction error using the principal component of the selected genes as the predictor. The components extracted by SPC are intended for prediction and not module identification. However, it is reasonable to consider using SPC for functional module discovery in the specific case that a single functional module influences the outcome since the genes selected would include the module genes and the principal component would load on the mutually correlated module genes. In this case, SPC exemplifies a two-step method that considers association with the outcome in the first step and coexpression in the second step, making it the reverse order compared to the WGCNA approach. For SPC, we used the package ‘superpc’ version 1.09 (Bair and Tibshirani, 2004).

Four simulation scenarios were considered: sample size of 100 with same sign coefficients, sample size of 100 with random sign coefficients, sample size of 400 with same sign coefficients and sample size of 400 with random sign coefficients. In the simple case of coefficients with the same sign, we let $\pi_{i}=1$ in (4) for all module genes so that all intramodular correlations resulted in the same sign. For random signs, we randomly selected each *π_i_* from {−1,1}. Each simulation scenario was repeated 200 times and bFMD, W, WGCNA and SPC were applied and compared under each simulation.

#### 3.1.3 Simulation results

We describe results for each method in terms of sensitivity, false discovery rate (FDR, where FDR was computed as 1 minus the average of the number of module variables discovered divided by the total number of variables selected), the distribution of detected module size and the Hamming distance between the detected module and the true module. The Hamming distance was normalized by the vector lengths so that 0 indicates perfect concordance and 100 indicates complete discordance.

**Fig. 3.**
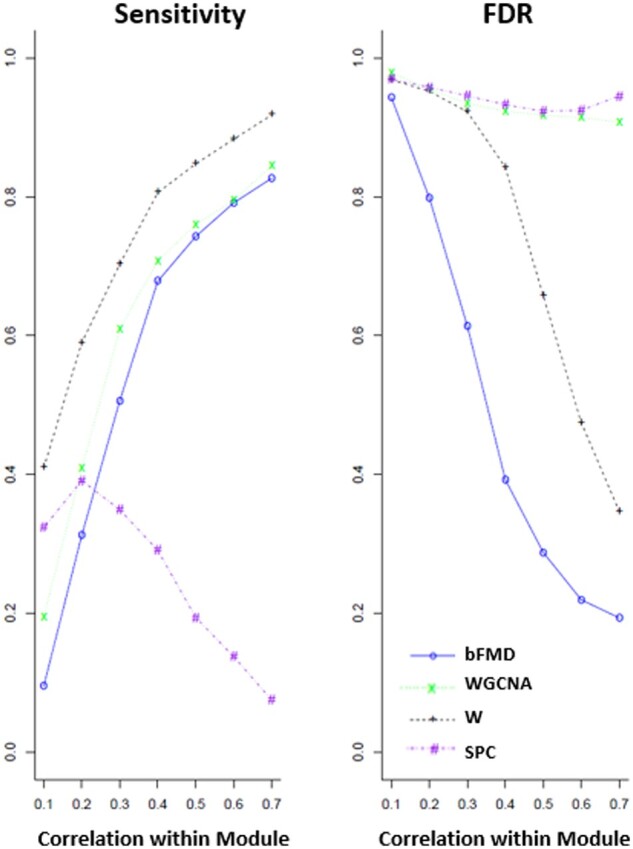
Sensitivity and FDR for SNR = 0.3 with sample size 100, generated with randomly signed edges in the graph. bFMD is our balance-based module discovery method, W is the method from Miecznikowski *et al.* (2016), WGCNA is an implementation of WGCNA using k-means for 6 clusters (Langfelder and Horvath, 2008) and SPC is sparse principal components (Bair *et al.*, 2006)

**Fig. 4.**
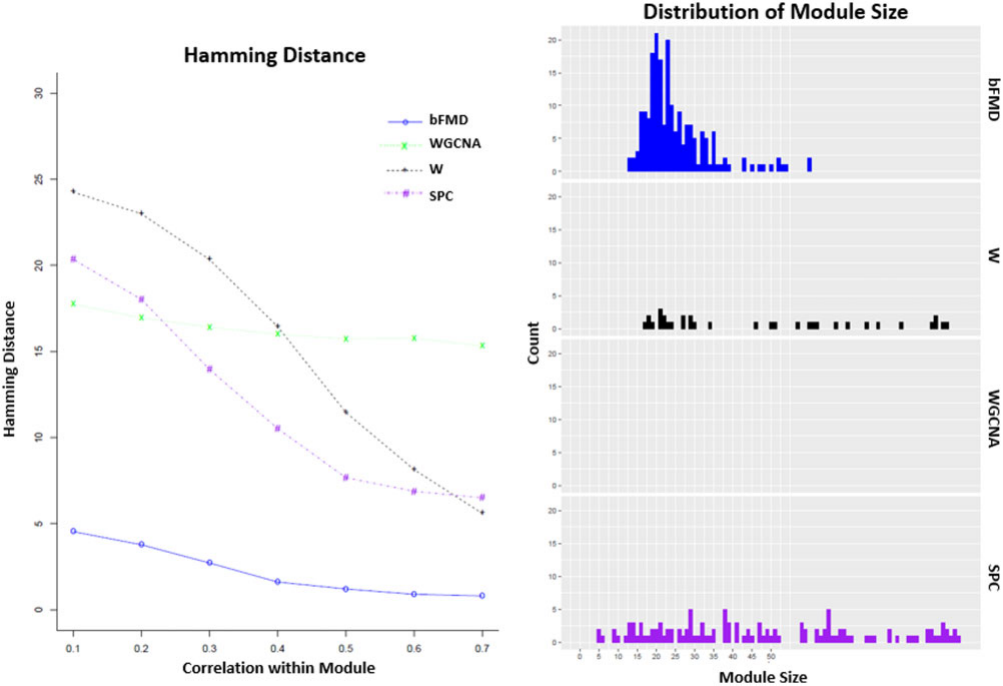
Hamming distance and distribution of module sizes for SNR = 0.3 and sample size 100, generated with randomly signed edges in the graph. bFMD is our balance-based module discovery method. W is the method from Miecznikowski *et al.* (2016), WGCNA is an implementation of WGCNA using k-means for 6 clusters (Langfelder and Horvath, 2008) and SPC is sparse principal components (Bair *et al.*, 2006). The module sizes are only shown up to 100. The interquartile ranges of module size for each method: bFMD (19, 28), W (149, 258), WGCNA (178, 192) and SPC (37, 108). Since the minimum module size for WGCNA is 155, its distribution is out of the range of this plot

Figures 3 and 4 were generated from a sample size of 100, SNR = 0.3, and had randomly signed edges. Figure 3 shows the sensitivity and FDR of the methods. The method W from Miecznikowski *et al.* (2016) selected the largest number of variables in the true module, followed by WGCNA and bFMD. The sensitivity of SPC plummets for higher intramodule correlations and bFMD by far results in the lowest FDR. The FDR for W is the most similar to bFMD, but it remains higher for all correlation values. The unacceptable FDR for WGCNA and SPC barely improves with higher intramodule correlation.

Figure 4 compares the Hamming distances and the distributions of the module sizes found by the methods. bFMD has by far the closest distance from the true module while also achieving smaller module sizes. Also, we see that module size for bFMD is tightly distributed about the true module size of 20. This is analogous to the case of a statistical estimator yielding low bias and low variance. Since the Hamming distance reflects both false positives and false negatives, the superiority of bFMD suggests that false discoveries heavily impact the distance measure due to the high dimensionality. WGCNA remains relatively unimproved by increasing the intramodule correlation, and SPC outperformed W.

The performance of the methods improves when SNR is increased to 0.5 due to the stronger signal, but the relative patterns remain similar, with the exception that the Hamming distance for W is greatly reduced. The interquartile ranges of module sizes become bFMD (21, 25), W (21, 168), WGCNA (177, 191) and SPC (30, 317).

For increased sample size of 400 and SNR = 0.3, the characteristics of bFMD and W tend to converge. The sensitivity of bFMD matches that of W for intramodular correlations over 0.2, and the FDR and Hamming distance of W match that of bFMD for correlations greater than 0.3. The Hamming distance for bFMD remains superior for all intramodule correlations.

To assess other module sizes, we ran similar simulations with the size of all modules increased to 50 with random signs and SNR = 0.3. Aside from the improved sensitivity in WGCNA, the patterns are consistent as in the simulations with module sizes of 20. Additionally, conclusions remain unchanged when repeating all of the simulations with entirely positive edges.

In summary, bFMD and W are superior to WGCNA and SPC. WGCNA suffers by not taking the association with *Y* into account when calculating clusters, which tend to be too large. SPC will select variables associated with *Y*, but those which are not correlated with other predictors will be missed. Only bFMD and W simultaneously consider both clustering and prediction criteria. W is more sensitive than bFMD, although the difference diminishes greatly for larger intramodule correlations. This is due to W producing larger modules, thus decreasing the probability of missing an important variable. However, the higher sensitivity in W also increases its FDR compared to bFMD since W overselects irrelevant variables. The k-means partition used by W is a crude surrogate for sparsity, but W and bFMD tend to converge with increased sample size and SNR, i.e. easier problems, since the underlying data structure operated on by the two methods is similar. Investigators should use the W approach if they are adverse to missing any important variable, and they should use bFMD if they are interested in getting the most accurate representation of the module.

### 3.2 Example

The fourth leading cancer amongst women in the USA may be attributed to Uterine Corpus Endometrial Carcinoma (UCEC), according to Levine *et al.* (2013). In 2013, there were 50 000 new diagnosed UCEC cases and the cause of approximately 8000 deaths in the USA. UCEC was selected for assessment in the Pan-Cancer project within The Cancer Genome Atlas (TCGA) since achieving a sufficient sample size would be made easy due to its high incidence. Mutation, copy number, gene expression, DNA methylation, MicroRNA and reverse-phase protein array were obtained from tumor samples across 545 donors, and the data were made publicly available in the TCGA database (Weinstein *et al.*, 2013).

For this example, we will focus on discovering the subset of genes from the gene expression dataset ($X^{*}$) that form a balanced module relating to percent tumor invasion (*Y*). The entire gene expression dataset within the TCGA-UCEC project contains 56 457 variables. To help reduce the amount of data in $X^{*}$, we will leverage prior research that shows mutations in MSH1, MSH2, MSH6 and PMS2 have been linked to Lynch Syndrome and ultimately Endometrial Cancers (Kempers *et al.*, 2011). Since MSH2 and MSH6 both reside on chromosome 2 and have been shown to be associated with one another (Schweizer *et al.*, 2001), we will focus on chromosome 2 for the analysis. Percent tumor invasion was selected as the outcome for this example since it associates with tumor grade and patient survival (Boronow *et al.*, 1984).

Ultimately, the analytical set resulted in 469 subjects, each with a percent tumor invasion outcome and 3811 genes to consider for the functional module. In notational form, we now have a matrix $X^{*}$ with 469 rows and 3811 variables (e.g. genes) and an outcome vector *Y* of length 469 (e.g. percent tumor invasion).

Analyses were done using bFMD, W, WGCNA and SPC. bFMD took the longest to execute at 20.1 min as compared to 1.8, 0.2 and 1.8 min for W, WGCNA and SPC, respectively. We describe the connectivity of estimated networks by the average correlation between module members, and the effect of the module on the outcome by the average correlation of module members with *Y*. For WGCNA, we estimated that there are six clusters by inspecting the eigenvalues of the correlation matrix (Fallah *et al.*, 2008). Table 1 summarizes the results, and we note that bFMD outperforms the other methods in every metric. While achieving the largest average module correlation and module effect, bFMD also returns the smallest module size, indicating a more tightly connected network. The value of Kappa for bFMD is 0.961, suggesting that the sparse eigenvector algorithm has produced a highly balanced module.

**Table 1.** TCGA-UCEC Transcription data: properties of estimated networks

	bFMD	W	WGCNA	SPC
Module size	187	680	795	248
Module correlation	0.23	0.19	0.05	0.12
Module effect	0.10	0.08	0.03	0.09

Table 2 shows the normalized Hamming distances between the variable selection vectors of the four methods. WGCNA produced the most different results compared to the other methods, which are likely attributed to its higher module size. bFMD produced the most similarly to SPC, likely due to the smaller module size from those two methods.

**Table 2.** TCGA-UCEC Transcription data: normalized Hamming distance between network variable selection vectors of bFMD, W, WGCNA and SPC

	bFMD	W	WGCNA	SPC
bFMD		6.5	8.3	5.6
W			12.4	9.4
WGCNA				13.0

**Fig. 5.**
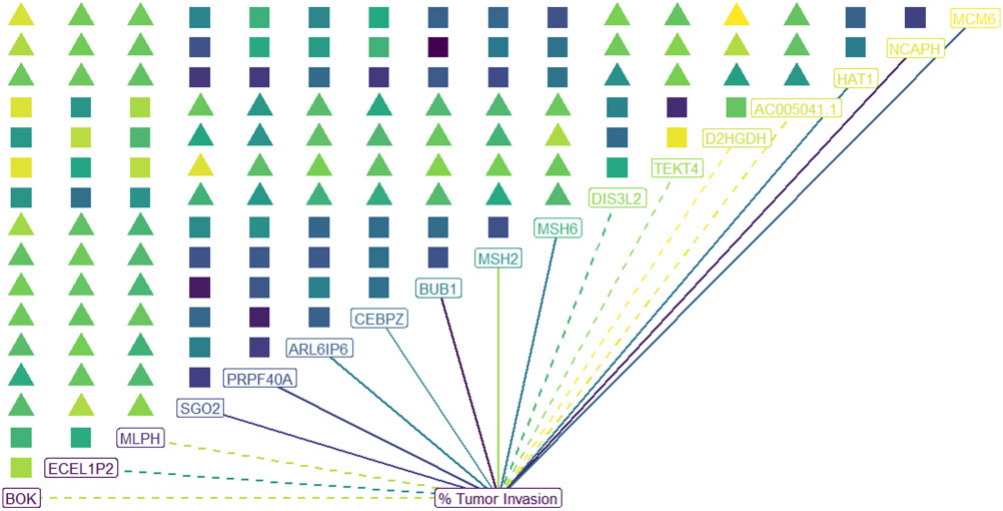
Symbolic representation of the 16 × 16 correlation matrix of module genes with strongest correlation with the biologically known genes MSH2 and MSH6. The color of the symbols corresponds to the absolute correlation for that pair. The symbol is a triangle when the correlation between the pair is negative, and the symbol is a square when the correlation is positive. The text color of the labeled genes corresponds to the principal component loading. The lines leading from a module variable to the outcome % Tumor Invasion are dotted when the correlation with invasion is negative and solid when the correlation is positive, and the line color indicates the magnitude of the correlation

The 187 features selected by bFMD included the MSH2 and MSH6 genes, which provide biological relevancy to the results. The W and WGCNA methods also selected the MSH2 and MSH6 genes, but they also selected many more genes which likely included noise due to the decreased module correlation and module effect. Despite the second smallest module size, moderate module correlation and module effects, SPC failed to select the MSH2 and MSH6 genes, weakening the biological interpretation of its selections. Figure 5 displays the selected module genes that are most negatively and positively correlated with MSH2 and MSH6.

From Figure 5, we see that MSH2 and MSH6 positively correlate with each other and with % Tumor Invasion, suggesting they are tumor promoters for UCEC. Conversely, we see that the selected gene TEKT4 was found to be negatively correlated with MSH2, MSH6 and % Tumor Invasion suggesting that TEKT4 is a tumor suppressor. This is consistent with prior research showing that TEKT4 loss relates to breast cancer metastasis (Ge *et al.*, 2021). The BOK gene has been shown to be a tumor suppressor for nonsmall-cell lung carcinoma (Moravcikova *et al.*, 2017), and it was selected by bFMD as negatively correlated with MSH2, MSH6 and % Tumor Invasion which also suggests it is a tumor suppressor for UCEC.

bFMD selected a much narrower set of genes as compared to the W method. To compare the gene ontology between these methods, we input the selections into DAVID (Huang *et al.*, 2009; Sherman *et al.*, 2009). Only 8 of the 187 genes selected by bFMD were unannotated within DAVID as compared to the 286 unannotated genes of the 680 selected by W. We clustered the annotated gene selections using DAVID’s Gene Functional Classification Tool which clusters genes based on similar gene-to-gene functional annotations. The goal of DAVID clustering is to combine biologically similar genes into a cluster. From this, DAVID identified 4 clusters across 18 genes from the selections by bFMD as compared to 7 clusters across 53 genes from the selections by W. Sixteen genes from the bFMD clusters were also contained within the W clusters. Additionally, the 4 clusters within bFMD corresponded to the top 4 clusters found within W where the ranking is according to enrichment score, a measure of cluster quality. In summary, bFMD identified a more biologically succinct set of genes as designed by the balance criteria.

## 4 Summary and discussion

The elements of an efficient biological mechanism may be expected to exhibit balanced effects which complement each other. The methods bFMD and W both operate on a matrix that highlights balanced sets of variables affecting an outcome variable as a positive submatrix. Of the two, bFMD most accurately identifies the set of module variables, as measured by the Hamming distance. Once the variables are extracted from a high-dimensional superset, detailed analysis of individual associations can be performed using existing methods for graphical model analysis (e.g. Edwards, 2000; Wermuth, 2012; Wermuth and Cox, 2013; Whittaker, 2009).

Our method is appropriate when linear relationships represent relevant aspects of the data. The theorems in this paper rely on the assumption of moderate conditional independence among variables and should be considered approximate for typical data sets. As with many statistical methods, strong multicollinearity potentially degrades performance. Future work could explore using a balance penalty in likelihood methods. For example, Net-Cox uses a prediction criterion to rate genes and ignores effect signs (Zhang *et al.*, 2013), but extensions could be explored to focus on causal consistency.

In general, there could be multiple independent functional modules $M_{1},M_{2},\ldots ,M_{K}$. There are many possible underlying models (Bianconi, 2018), depending on the postulated relation among the blocks. Sparse analysis for multiblock models has special complications even in the simple case of independent blocks (Mackey, 2009). Extending balance to sequential blocks of different variable types, e.g. integrating genotype and gene expression by modeling expression as intermediate between genotype and outcome, will provide a rich source of problems for future work.

## Supplementary Material

vbab018_Supplementary_Data

## Data Availability

The code to simulate functional modules and perform the bFMD method may be found on Github at https://github.com/lorinmil/bFMD. The data from the Example section may be downloaded from the TCGA-UCEC project.
