# Supplementary material for: Balanced Functional Module Detection in genomic data
Source: Bioinform Adv. 2021 Sep 16;1(1):vbab018. doi: 10.1093/bioadv/vbab018 (PMC9710612; doi:10.1093/bioadv/vbab018)
Supplement: vbab018_Supplementary_Data [file vbab018_supplementary_data.pdf]

1 Appendix

1.1 Definitions

The preliminary definitions are described below.

Definition 1.1 ***Balanced Sign Graph***. *A signed graph G is "balanced" if and only if for every two nodes of G, all paths joining them have the same sign (Chartrand (1977), Ch. 8; Harary (1953)).*

Definition 1.2 ***Balanced Functional Network ($G \cup Y$).*** *$G \cup Y$ is a "balanced functional module" when the following are satisfied.*

1. *G is balanced.*
2. *G is connected: at least one path connecting each pair of elements within G exists.*
3. *Every node of G is connected to Y by a semi-directed path with at least one directed edge.*
4. *For a given node $i \in G$, all $i - Y$ paths have the same sign.*

1.2 Theorems and Lemmas

The proofs for the theorems and lemmas are described below.

Theorem 1.1 *For balanced functional network $G \cup Y$, let $\alpha \cup Y$ equal a semi-directed $i \to Y$ path, γ equal an $i - j$ path in G, and $\tau \cup Y$ equal a semi-directed $j \to Y$ path. Then $w^{\alpha \cup Y} \cdot w^{\gamma} \cdot w^{\tau \cup Y} > 0$.*

Proof 1.1 Note that $w^{\gamma} \cdot \beta_{Y|j.X/j}$ equals the weight of an $i - Y$ path, so $sign(w^{\alpha \cup Y}) = sign(w^{\gamma} \cdot \beta_{Y|j.X/j}) = sign(w^{\gamma}) \cdot sign(\beta_{Y|j.X/j})$. However, $\beta_{Y|j.X/j}$ equals the weight of the directed $j \to Y$ edge which is also a $j - Y$ path. Hence, $sign(\beta_{Y|j.X/j}) = sign(w^{\tau \cup Y})$. Now, $sign(w^{\alpha \cup Y}) = sign(w^{\gamma}) \cdot sign(w^{\tau \cup Y})$, and the product of the three terms $w^{\alpha \cup Y}, w^{\gamma}, w^{\tau \cup Y}$ must be positive.

Lemma 1.2 *For a balanced graph G, the sign of an $i - j$ walk is the sign of every $i - j$ path in G.*

Proof 1.2 Let γ be an $i-j$ walk in G. If γ is not a path then there exists a cycle where a repeated node is connected by a path within γ. Since every cycle in a balanced graph is positive (Harary (1953); Chartrand (1977), Theorem 8.2), the cycle can be excised from the walk without changing the sign of the walk. This process can be continued until ultimately an $i-j$ path with the same sign as γ remains.

Theorem 1.3 *For a balanced graph G and corresponding covariance matrix $\Sigma = (\sigma_{ij})$, if we assume moderate conditional dependence then the sign of σ_{ij} is equivalent to the sign of every $i-j$ path in G.*

Proof 1.3 The sign of σ_{ij} equals the sign of the summed $i-j$ walk weights in G, and each $i-j$ path contains the same sign as the $i-j$ walks.

Theorem 1.4 *For a balanced functional network $G \cup Y$ with G satisying the conditions of Theorem 1.3:*

1. *$Cov(i,Y)$ is a positively weighted sum of all $i-Y$ walk weights*
2. *The sign of $Cov(i,Y)$ equals the sign of every $i-Y$ path.*
3. *$Cov(i,Y) \neq 0$.*

Proof 1.4 By Proposition 5.5.1 of (Whittaker (2009)):

$$\begin{aligned}
Cov(Y,i) &= Cov(Y,i|X/i) + Cov(Y,X/i)Var(X/i)^{-1}Cov(X/i,i) \\
&= \beta(Y|i.X/i)d_i^{-1} + \Sigma_{j\neq i}\beta(Y|j.X/i,j)Cov(i,j) \\
&= \beta(Y|i.X/i)d_i^{-1} + \Sigma_{j\neq i}\beta(Y|j.X/i,j)d_i^{-1/2}d_j^{-1/2}w^*(i,j) \\
&= \beta(Y|i.X/i)d_i^{-1} + \Sigma_{j\neq i}\psi(i,j)d_i^{-1/2}d_j^{-1/2}w^*(i,j)\beta(Y|j.X/j),
\end{aligned}$$

where $\psi(i,j) = \beta(Y|j.X/i,j)/\beta(Y|j.X/j)$ corresponds to the ratio of the partial regression coefficient for x_j in the regression model with predictor set X/i to the coefficient for x_j in the model including x_i as a predictor. The d_k weights are positive since they are variances. $\psi(i,j)$ is positive if adding x_i to the predictors does not change the sign of the regression coefficient for x_j. We take the condition of moderate

conditional dependence required for Theorem 1.3 to imply that such sign change of coefficients will not occur. The first term of the last equation is proportional to the direct $i \to Y$ edge, and the summation in that equation is a positively weighted sum of indirect $i \to Y$ walks passing through other nodes in G. This proves the first statement of the theorem. By Lemma 1.2, $w^*(i,j)$ equals the sum of walk weights with the same sign as every $i-j$ path, so the sign of $w^*(i,j)\beta(Y|j.X/j)$ equals the sign of any $i-Y$ path with weight $w(i,j)\beta(Y|j.X/j)$. This proves the second statement. The third statement immediately follows because $Cov(i,Y)=0$ implies that the sign of every $i-Y$ path is zero, contradicting statement 3 of Definition 1.2.

Theorem 1.5 *If the graph $G \cup Y$ within X is a balanced functional module, then*

$$M = diag(Cov(X,Y))Var(X)diag(Cov(X,Y)) \tag{1}$$

is a positive matrix (i.e. all elements of M are > 0).

Proof 1.5 By Theorems 1.3 and 1.4, $sign(Cov(x_i,Y))$ equals the sign of any $i-Y$ path α, $sign(Cov(x_j,Y))$ equals the sign of any $j-Y$ path τ, and $sign(Cov(i,j))$ equals the sign of any $i-j$ path γ in G. Thus, for every i,j pair, $sign(Cov(x_i,Y)Cov(i,j)Cov(x_p,Y)) = sign(w_{iY}^{\alpha} \cdot w_{ij}^{\gamma} \cdot w_{jY}^{\tau}) > 0$ by Theorem 1.1.

Lemma 1.6 *With balanced A and B as defined above, all paths in the signed graph G connecting nodes in the same set are positive and all paths connecting a node in A to a node in B are negative.*

Proof 1.6 If a path in G connects nodes in A, then it must have zero or an even number of negative edges from A to B. Since all edges in A are positive, the path must be positive. Likewise for B. If a path in G connects a node in A to a node in B, then it must have zero or an odd number of negative edges from A to B. Since edges within A and B are positive, the path must be negative.

Theorem 1.7 *For a balanced functional network $G \cup Y$, G can be partitioned into two sets of variables A and B such that elements of A are positively correlated, elements of B are positively correlated, and correlations between A and B are negative. All elements of the same set have correlations with Y of the same sign, which is opposite for the two sets.*

Proof 1.7 By Lemma 1.6, the balanced graph G can be partitioned into sets A and B with positive paths connecting the nodes in the same set, and negative paths from one set to the other. By Theorem 1.3, variables in A are positively correlated, variables in B are positively correlated, and correlations between A and B are negative, proving the first statement. To prove the final statement, note that for a functional module $G \cup Y$ there must be at least one variable j in G with an edge $e : j \to Y$. Arbitrarily assume $j \in A$. By property 2 of Definition 1.2, for each $i \in A$ there is an $i - j$ path. By Lemma 1.6, the $i - j$ path is positive and so the $i - j \to Y$ path has the sign of e. By property 4 of Definition 1.2, all $i - Y$ paths for $i \in A$ have the same sign as e, and by Theorem 1.4, $Cov(i, Y)$ has the sign of e for all $i \in A$. Since G is connected, if $k \in B$ then there exists a negative path to j by Lemma 1.6, and hence the $k - j \to Y$ path has an opposite sign from e. Thus, by Theorem 1.4, all variables in B have correlation with Y of sign opposite from e, proving the second statement of the theorem.

1.3 Tuning for Sparse Principal Component Analysis

The sparse principal component method from Sigg and Buhmann (2008) specifies sparsity by inputting the number of nonzero elements, k. We calculate the positive eigenvector for all feasible k (e.g. module sizes), and we select the sparsity setting k resulting in the optimally balanced solution. Balance may be quantified through the balance density $\mathcal{M}(k) = \Sigma_{i \neq j} m_{ij} / \Sigma_{i,j} abs(m_i j)$ for a matrix in the form of (1). This can be motivated by an example where the matrix elements corresponding to the module of size p have values $r > 0$, and the non-module elements have values of 0. Thus, an effective sparse positive eigenvector algorithm will provide solutions

$$\begin{aligned} \mathcal{M}(k) &= k(k-1)r/(k(k-1)r + k), k \leq p \\ &= p(p-1)r/(p(p-1)r + k), k > p. \end{aligned}$$

It then follows that $M(k+1) - \mathcal{M}(k) = \frac{r}{(1+(k-1)r)(1+kr)} > 0$ when $k \leq p$, and $\mathcal{M}(k+1) - \mathcal{M}(k) < 0$ when $k > p$. This suggests that $\mathcal{M}(k)$ increases with respect to k for effective values of k. The decrease

enlarges when negative elements are included. Thus, the maximum balance density may be used to tune the module size k in the Sigg-Buhmann algorithm for sparse principal component analysis.

1.4 Details on Simulation

We consider a SIM represented by the linear model for gene expression

$$\begin{aligned} x_i &= \pi_i \beta x_0 + \varepsilon_i, i = 1, \ldots, t \\ x_0 &= \varepsilon_0 \end{aligned}$$

where $\beta > 0$, $\pi_i \in \{-1, 1\}$ and the ε_i, $i = 0, 1, \ldots, t$ are independent errors with mean 0 and variance σ_ε^2. The covariance of all pairs of genes in this system are nonzero. The covariation among the t observed module genes are driven by a latent unobserved *hub*, x_0. Letting x_i and x_j be two observed (non-hub) module genes, $Cov(x_i, x_j) = \pi_i \pi_j \beta^2 \sigma_\varepsilon^2$and $Cov(x_i, x_0) = \pi_i \beta \sigma_\varepsilon^2$. The non-hub variances equal $\sigma_\varepsilon^2(1 + \beta^2)$, and the correlation between observed module genes x_i and x_j is $r_{x_i, x_j} = \frac{\pi_i \pi_j \beta^2}{1+\beta^2}$.

We model the functional aspect of the pathway by letting the hub x_0 determine an outcome variable y by the regression function

$$y = \alpha x_0 + \delta,$$

where $\alpha > 0$ without loss of generality. Letting the variance of the error term δ in (1.4) be σ_δ^2, $Cov(y, x_i) = \pi_i \alpha \beta \sigma_\varepsilon^2$ for a non-hub gene x_i, $Cov(y, x_0) = \alpha \sigma_\varepsilon^2$, $Var(y) = \alpha^2 Var(x_0) + \sigma_\delta^2 = \alpha^2 \sigma_\varepsilon^2 + \sigma_\delta^2$, and $cor(y, x_i) = \frac{\alpha}{\sqrt{\alpha^2 \sigma_\varepsilon^2 + \sigma_\delta^2}} \frac{\pi_i \beta \sigma_\varepsilon^2}{\sqrt{\sigma_\varepsilon^2 (1+\beta^2)}}$.

1.5 Hamming Distance Calculation

Hamming distance is computed by defining q to be the binary vector which indicates selected variables by a procedure, and let q_0 indicate the true module variables. The raw Hamming distance is the number of positions in disagreement across the two vectors. We report the distance normalized by the length of the

vectors and express the proportion as a percentage, where 0 means perfect concordance and 100 means complete discordance. Hamming distance increases with both missed variables and false inclusions, making it a natural measure for the similarity between the computed module and the true module.

1.6 TCGA Data Extraction

Upon initial query of the TCGA-UCEC data, the clinical dataset (containing the information on percent tumor invasion) had 596 records and the gene expression dataset had 587 records. To clean the data, variable values within the gene expression dataset were averaged across any duplicate subject records, only the intersecting subject IDs across both the clinical and gene expression datasets were considered, and any subjects with missing information on percent tumor invasion were excluded. Subsequently, only the genes from chromosome 2 were considered and any genes with 0 variance were excluded. Furthermore, since the percent tumor invasion should range from 0 to 100, any patients with values outside of this range will be removed.

References

Chartrand, G. (1977), *Introductory Graph Theory*, Dover, New York.

Harary, F. (1953), 'On the Notion of Balance of a Signed Graph', *The Michigan Mathematical Journal* **2**, 143–146.

Sigg, C. D. and Buhmann, J. M. (2008), Expectation-Maximization for Sparse and Non-Negative PCA, *in* 'Proceedings of the 25th International Conference on Machine Learning', ACM, New York, p. 960967.

Whittaker, J. (2009), *Graphical Models in Applied Multivariate Statistics*, Wiley, New York.
